# Association and predictability of major perioperative cardiovascular adverse events and elevated neutrophil percentage-to-albumin ratio in patients with stable coronary artery disease undergoing non-cardiac surgery

**DOI:** 10.3389/fcvm.2025.1623731

**Published:** 2025-09-19

**Authors:** Haodong Jiang, Jia Zhu, Congying Wang, Xiaojun Xia, Runzhe Wu, Feiyu Chen, Yongquan Niu, Yunpeng Jin

**Affiliations:** Department of Cardiology, the Fourth Affiliated Hospital of School of Medicine, and International School of Medicine, International Institutes of Medicine, Zhejiang University, Yiwu, China

**Keywords:** neutrophil percentage-to-albumin ratio, major adverse cardiovascular events, coronary artery disease, noncardiac surgery, machine learning

## Abstract

**Objective:**

To evaluate the utility of the preoperative neutrophil percentage-to-albumin ratio (*N*PAR) for predicting perioperative major adverse cardiovascular events (MACE) in patients with stable coronary artery disease (SCAD) undergoing non-cardiac surgery.

**Methods:**

In this retrospective cohort study, we included all adult SCAD patients who underwent non-cardiac surgery at the Fourth Affiliated Hospital of Zhejiang University School of Medicine between October 2020 and October 2024. The primary endpoint was the occurrence of MACE during the perioperative period, defined as a composite of all-cause mortality, cardiac arrest, myocardial infarction, heart failure, or stroke occurring intraoperatively or during the postoperative hospital stay. We used multivariable logistic regression to assess the independent association between NPAR and MACE risk. To explore potential nonlinearity, we fitted smooth curves and performed threshold-effect analysis. Mediation analysis quantified the proportion of the NPAR–MACE relationship explained by estimated glomerular filtration rate (eGFR). Incremental predictive value was evaluated by comparing the area under the receiver operating characteristic curve (AUC), net reclassification improvement (NRI), and integrated discrimination improvement (IDI) before and after adding NPAR to established risk models. Feature selection was conducted using the Boruta algorithm, and predictive performance was further validated with an XGBoost model interpreted via Shapley Additive Explanations (SHAP).

**Results:**

Of 1,771 patients, 90 (5.1%) experienced MACE. The MACE subgroup had a higher mean NPAR than those without events (19.4 ± 5.3 vs. 15.9 ± 3.5; *P* < 0.001). Each 1-unit increase in NPAR was associated with a 20% higher risk of MACE (OR 1.20; 95% CI 1.10–1.30). A J-shaped relationship emerged, with an inflection point at NPAR 13.7 (P_threshold = 0.005). eGFR mediated 8.4% of the NPAR–MACE association. NPAR alone yielded an AUC of 0.721. Adding NPAR to the Revised Cardiac Risk Index raised the AUC from 0.679–0.755 (NRI 0.599; IDI 0.035; all *P* < 0.01). The XGBoost model achieved an AUC of 0.773, and SHAP analysis identified NPAR as the most influential predictor.

**Conclusions:**

Preoperative NPAR is an independent, readily available predictor of perioperative MACE in SCAD patients. Incorporation of NPAR into existing risk models significantly enhances predictive accuracy and may inform targeted perioperative management strategies.

## Introduction

Each year, millions of patients worldwide undergo non-cardiac surgery (NCS), with over 18% of cases involving individuals with stable coronary artery disease (SCAD) (1). Among SCAD patients undergoing NCS, 5.7%–10.0% experience major adverse cardiovascular events (MACE) during the perioperative period (2–4), a rate significantly higher than the 2.5%–3.0% observed in the general surgical population (5). MACE—including cardiac arrest, myocardial infarction (MI), heart failure (HF), and stroke (6)—represents a major contributor to perioperative morbidity and mortality, imposing a considerable social and economic burden. Therefore, accurate prediction of perioperative MACE risk is crucial. It enables clinicians to comprehensively assess surgical risks, tailor perioperative management plans, facilitate informed shared decision-making with patients, and optimize the allocation of healthcare resources.

The development of postoperative cardiovascular events is influenced by multiple factors, among which inflammation has recently emerged as a potential driving mechanism. Inflammatory responses contribute to cardiovascular injury through various pathways. Chronic inflammation can impair endothelial function and promote the formation of atherosclerotic plaques. Inflammatory cytokines such as interleukin-6 (IL-6) and tumor necrosis factor-alpha (TNF-α) enhance platelet aggregation and increase plaque vulnerability (7–9). Additionally, oxidative stress-induced inflammatory cascades may directly damage cardiomyocytes. Systemic inflammation is closely associated with the progression of atherosclerosis, alterations in hemostasis, the presence and severity of coronary artery disease (CAD), and the development of acute coronary thrombosis (10, 11). Collectively, these mechanisms promote plaque destabilization, thrombogenesis, and microvascular dysfunction, ultimately leading to MACE. Recent studies have indicated that surgical procedures significantly exacerbate inflammatory responses. Surgical trauma elicits a physiological stress response—including heightened sympathetic nervous system activity, catecholamine release, and hemodynamic fluctuations—that not only increases myocardial oxygen demand but also triggers systemic inflammation characterized by the release of proinflammatory cytokines and activation of immune cells. These processes may impair endothelial function, destabilize plaques, and induce microvascular dysfunction, thereby accelerating myocardial injury (12). Thus, evaluating the preoperative inflammatory status is critical for predicting perioperative cardiovascular risk in patients with SCAD.

Inflammatory biomarkers provide a quantitative means of assessing systemic inflammation, and recent studies have shown that inflammatory biomarkers are useful prognostic tools for cardiovascular disease and that their integration into risk stratification models may improve their predictive accuracy (13, 14). Several inflammation-based indicators—such as high-sensitivity C-reactive protein (hs-CRP), the systemic immune-inflammation index (SII), and the advanced lung cancer inflammation index (ALI)—have been shown to correlate with prognosis in patients with acute coronary syndrome (ACS) (15–18). Recently, the NPAR, a novel composite biomarker that integrates neutrophil percentage and serum albumin level, has been proposed as a sensitive indicator of persistent inflammation associated with cardiovascular injury (19). Owing to its cost-effectiveness and accessibility, NPAR has demonstrated prognostic value in patients with cancer and myocardial infarction (20). However, no studies to date have evaluated the predictive value of NPAR for perioperative cardiovascular events in patients with SCAD. Therefore, this study aims to investigate the association between preoperative NPAR and the incidence of MACE in patients with SCAD undergoing NCS, and to explore the prognostic utility of NPAR in this clinical setting.

## Methods

### Study design and participants

This retrospective study included data from patients aged 18 years or older with known SCAD who underwent NCS at the Fourth Affiliated Hospital, Zhejiang University School of Medicine (FAHZU), between October 1, 2020, and October 31, 2024. The study was conducted in accordance with the Declaration of Helsinki and was approved by the Institutional Review Board (IRB) and Ethics Committee of FAHZU (Approval No. K2024222). Due to the retrospective nature of the study, informed consent was waived in accordance with national regulations and institutional policies. Patient data were extracted from the hospital's electronic medical record system using the International Classification of Diseases, 10th Revision (ICD-10). All adult patients discharged with a diagnosis of coronary artery disease (CAD, ICD-10 codes: I21–I25) and who underwent surgery during hospitalization within the study period were identified.

**Figure 1:**
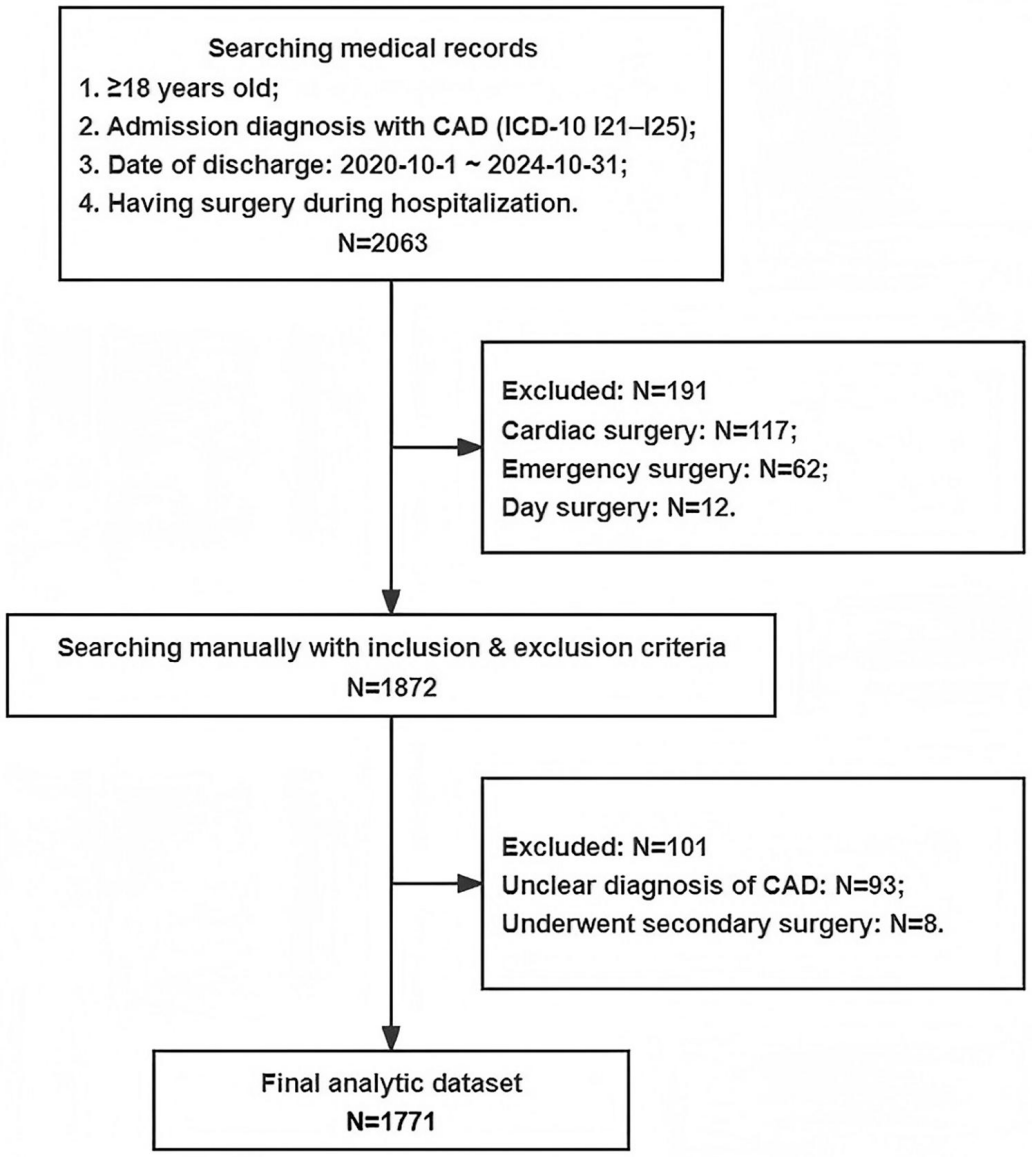
Flowchart of participant selection.

To ensure scientific validity, internal consistency, clinical relevance, and data quality, we applied strict inclusion and exclusion criteria. Figure 1 shows details of the screening criteria for the study population. After applying these criteria, a total of 1,771 patients were included in the final analysis. SCAD was defined according to the American College of Cardiology (ACC)/American Heart Association (AHA) guidelines for perioperative cardiovascular evaluation in elective non-cardiac surgery. A diagnosis of SCAD was made if any of the following conditions were met: coronary angiography showing >50% stenosis; myocardial infarction occurring more than 3 months before enrollment; positive exercise stress test; positive myocardial perfusion electrocardiogram; or typical angina symptoms accompanied by evidence of myocardial ischemia (21, 22). All patients underwent standard preoperative assessment.

### Assessment of perioperative MACEs

The primary endpoint was a composite of MACEs occurring intraoperatively or during the postoperative hospital stay. This included all-cause mortality, resuscitated cardiac arrest, MI, HF, and stroke. Cardiac arrest was defined as a loss of circulation requiring resuscitation with chest compressions, defibrillation, or both (23). MI was defined as acute myocardial injury with clinical evidence of acute myocardial ischemia. diagnosed based on the detection of an increase or decrease in cardiac troponin values, with at least one value greater than the 99th percentile upper reference limit and at least one of the following: (a) symptoms of myocardial ischemia; (b) new ischemic ECG changes; (c) development of pathological Q waves; (d) imaging evidence of new loss of viable myocardium or new regional wall motion abnormality in a pattern consistent with an ischemic etiology; and (e) identification of a coronary thrombus by angiography or autopsy. Routine troponin testing was not performed for any enrolled patients; troponin levels were ordered only when the treating physician suspected MI based on clinical presentation or electrocardiographic changes, in accordance with standard clinical practice (24). HF was diagnosed primarily based on active clinical symptoms or physical findings, such as dyspnea, orthopnea, peripheral edema, jugular venous distension, rales, a third heart sound, or chest radiographic evidence of pulmonary vascular redistribution or pulmonary edema (25). Stroke was diagnosed by a neurology consultant based on new neurological deficits confirmed by imaging studies (26).

### Assessment of the NPAR

Neutrophil percentage and serum albumin levels were obtained from routine preoperative laboratory examinations. All preoperative laboratory assessments were conducted within a 30-day window preceding the surgery. To ensure the analysis was based on the most clinically relevant data, extraneous measurements were excluded. When multiple test results were available for a patient during this period, the value obtained closest to the date of surgery was selected for analysis. The NPAR was calculated using the following formula (27):

$$NPAR=\frac{Neutrophil\;percentage\;(\text{\%})\;*\;100}{Albumin\;(g/dl)}$$

Based on previous studies, participants were stratified into three groups according to NPAR tertiles: tertile 1 (reference group), tertile 2, and tertile 3.

### Covariates

Medical records were further manually screened based on the inclusion and exclusion criteria. Clinical data were extracted from the electronic medical record system. Covariates were selected based on previous research findings and clinical expertise, and included demographic characteristics, comorbidities, preoperative laboratory results, American Society of Anesthesiologists (ASA) classification, type of surgery, anesthesia method, length of hospital stay, and perioperative events. ASA class III and IV were defined as patients with severe systemic disease (class III) and patients with severe systemic disease that poses a constant threat to life (class IV) (28), respectively. Since ASA classification was assessed preoperatively, the evaluators were blinded to patient outcomes.

### Statistical analysis

The few covariates were interpolated using the Random Forest algorithm to minimize estimation bias. Missing values were imputed via the missForest package; Supplementary Material provide both the raw and imputed datasets. The proportion of missing data was <15%, and the distribution of missingness is presented in Supplementary Figure S1. Continuous variables were expressed as mean ± standard deviation (SD) and median with interquartile range, depending on their distribution. For variables with a normal distribution, comparisons between groups were conducted using the two-sample *t*-test; otherwise, the Mann–Whitney *U* test was applied. Categorical variables were presented as frequencies and percentages, and group differences were assessed using the Pearson chi-square test. Multivariable logistic regression was used to explore the association between NPAR and outcomes. NPAR values were divided into tertiles, with the lowest tertile (Low) serving as the reference group. Three models were constructed to evaluate these associations: Model 1 was unadjusted; Model 2 was adjusted for age, gender, BMI, hypertension, and diabetes; Model 3 included all potential confounders. The median value of each tertile was treated as a quasi-continuous variable in the regression model to test for trend (*P* for trend). To further investigate the relationship between NPAR and the outcome, we used a generalized additive model (GAM) and smooth curve fitting to identify potential nonlinear associations. Piecewise linear regression analysis was then performed to determine the inflection point, followed by a two-piecewise linear regression to analyze threshold effects on either side of the inflection point. To evaluate whether eGFR mediated the relationship between NPAR and perioperative MACE, we performed mediation analysis. The indirect, direct, and total effects were calculated, and the mediation proportion was defined as (indirect effect)/(indirect + direct effect) × 100%. Mediation was considered statistically significant only if the total, indirect, and mediation effects were all significant and positive. Mediation analysis was conducted using the mediation effect module in EmpowerStats software. The Revised Cardiac Risk Index (RCRI), commonly used in clinical settings to assess cardiac risk in non-cardiac surgery, was used as a reference. Model discrimination was evaluated using the ROC curve, area under the ROC curve (AUC), C-statistic, NRI, and IDI. The DeLong test was used to compare AUC values between models with and without NPAR. In addition, we combined the NPAR with the RCRI model to create a clinical diagnostic nomogram. To identify important predictors, we used the Boruta algorithm, which evaluates the importance of each variable by comparing its Z-score to that of shadow features. Variables with significantly higher Z-scores than their shadow counterparts were considered “important” (green zone), while those without significant differences were deemed “unimportant” (red zone) (29). NPAR was also included in this framework as a predictive feature. Key variables identified were used to develop a perioperative MACE risk prediction model. The dataset was randomly split into a training set and a validation set at a 7:3 ratio. Model training was performed using the Extreme Gradient Boosting (XGBoost) algorithm, with hyperparameters optimized via 5-fold cross-validation repeated 10 times. The primary objective was to maximize the AUC to enhance predictive performance. The SHAP method was employed to quantify the contribution and impact of each feature on the model's predictions. Higher SHAP values indicated greater importance in the model's decision-making process, helping interpret the influence of individual predictors. Decision curve analysis (DCA) was conducted to assess clinical utility, and calibration curves were generated to evaluate the model's predictive accuracy. Additionally, sensitivity analyses were performed. Patients with missing data were excluded to assess the robustness of the primary findings. All statistical tests were two-sided, with a significance threshold of *P* < 0.05. Statistical analyses were conducted using R software (version 4.3.2) and EmpowerStats (version 6.0).

## Result

### Baseline characteristics

Table 1 summarizes the demographic details of the participants. In this study, a total of 1,681 non-MACE patients and 90 MACE patients were included. Among the MACE group, 57 patients (63.3%) were male. The mean NPAR in the control group was 15.9 ± 3.5, compared to 19.4 ± 5.3 in the MACE group, a statistically significant difference (*p* < 0.001). MACE patients were generally older, had lower BMI, and a higher prevalence of comorbidities such as hypertension and dialysis history. They also exhibited higher rates of stroke and atrial fibrillation. The incidence of ASA class III (71.1% vs. 52.6%, *p* < 0.001) and ASA class IV (7.8% vs. 0.8%, *p* < 0.001) was significantly elevated in the MACE group. Additionally, neutrophil count and serum creatinine levels were significantly higher in MACE patients, while BMI, hemoglobin, albumin and eGFR were notably lower. Significant univariate predictors were subsequently included in multivariate analysis.

**Table 1:** Baseline characteristics of participants.

Variables	Non-MACEs	MACEs	*P*-value
	(*n* = 1,681)	(*n* = 90)	
Demographic			
Age (years)	68.9 ± 9.3	75.2 ± 9.3	<0.001
BMI (kg/ m^2^)	24.4 ± 3.4	23.5 ± 3.6	0.014
Gender			0.663
Male	1,026 (61.0%)	57 (63.3%)	
Female	655 (39.0%)	33 (36.7%)	
Comorbidities			
Hypertension			0.001
No	510 (30.3%)	13 (14.4%)	
Yes	1,171 (69.7%)	77 (85.6%)	
Diabetes			0.834
No	1,194 (71.0%)	63 (70.0%)	
Yes	487 (29.0%)	27 (30.0%)	
Stroke			<0.001
No	1,514 (90.1%)	70 (77.8%)	
Yes	167 (9.9%)	20 (22.2%)	
Dialysis			<0.001
No	1,658 (98.6%)	84 (93.3%)	
Yes	23 (1.4%)	6 (6.7%)	
COPD			0.243
No	1,626 (96.7%)	85 (94.4%)	
Yes	55 (3.3%)	5 (5.6%)	
Ischemic heart disease			0.195
No	771 (45.9%)	35 (38.9%)	
Yes	910 (54.1%)	55 (61.1%)	
Myocardial infarction			0.007
No	1,578 (93.9%)	78 (86.7%)	
Yes	103 (6.1%)	12 (13.3%)	
Myocardial infarction			<0.001
No	1,602 (95.3%)	65 (72.2%)	
Yes	79 (4.7%)	25 (27.8%)	
Atrial fibrillation			<0.001
No	1,597 (95.0%)	73 (81.1%)	
Yes	84 (5.0%)	17 (18.9%)	
Valvular heart disease			0.427
No	1,660 (98.8%)	88 (97.8%)	
Yes	21 (1.2%)	2 (2.2%)	
PTCA			0.114
No	872 (51.9%)	39 (43.3%)	
Yes	809 (48.1%)	51 (56.7%)	
CABG			0.452
No	1,642 (97.7%)	89 (98.9%)	
Yes	39 (2.3%)	1 (1.1%)	
Preoperative blood tests			
Leukocyte (×10^9^/L)	6.2 ± 2.1	6.8 ± 3.1	0.096
Neutrophil (×10^9^/L)	4.0 ± 1.9	4.9 ± 3.0	<0.001
Hemoglobin (g/L)	130.5 ± 18.4	116.9 ± 21.7	<0.001
LDL-C (mmol/L)	1.9 ± 0.7	2.0 ± 0.7	0.22
ALB (g/L)	40.3 ± 4.2	36.9 ± 4.9	<0.001
ALT (U/L)	25.7 ± 26.0	26.9 ± 20.7	0.871
AST (U/L)	25.8 ± 20.5	31.7 ± 30.8	0.532
Creatinine (*μ*mol/L)	87.4 ± 84.4	135.8 ± 179.7	<0.001
Uric acid (μmol/L)	323.7 ± 94.5	328.8 ± 123.2	0.714
eGFR (ml/min/1.73 m^2^)	89.5 ± 27.8	74.2 ± 32.0	<0.001
D-dimer (mg/L FEU)	1.3 ± 3.5	2.5 ± 3.3	<0.001
ASA class			<0.001
II	783 (46.6%)	19 (21.1%)	
III	885 (52.6%)	64 (71.1%)	
IV	13 (0.8%)	7 (7.8%)	
General anesthesia			0.671
No	445 (26.5%)	22 (24.4%)	
Yes	1,236 (73.5%)	68 (75.6%)	
Types of surgery			<0.001
General Abdominal	212 (12.6%)	27 (30.0%)	
General Non-abdominal	146 (8.7%)	4 (4.4%)	
Neurological	40 (2.4%)	9 (10.0%)	
Orthopedic	607 (36.1%)	41 (45.6%)	
Thoracic	192 (11.4%)	3 (3.3%)	
Urologic	316 (18.8%)	3 (3.3%)	
Other	168 (10.0%)	3 (3.3%)	
DOS (min)	87.3 ± 63.6	112.8 ± 89.3	0.006
NPAR	15.9 ± 3.5	19.4 ± 5.3	<0.001

### Association between NPAR and perioperative MACEs

**Figure 2:**
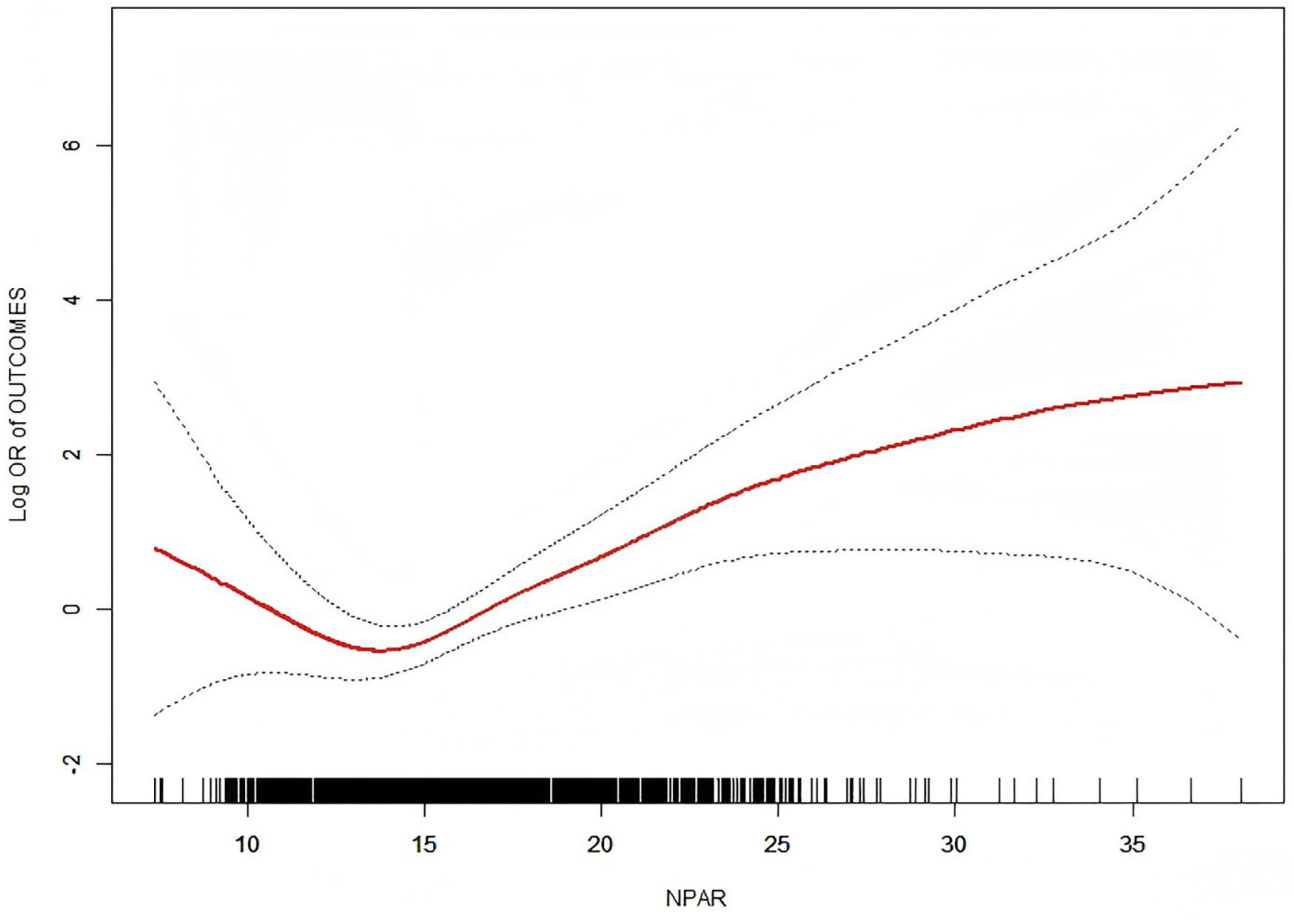
Density-dose response relationship between NPAR and perioperative MACEs. The area between the upper and lower dotted lines is represented as 95% CI.

Multivariate logistic regression analysis demonstrated a positive association between NPAR and the incidence of perioperative MACEs. This relationship remained significant even after adjusting for all potential confounders [odds ratio (OR): 1.2; 95% confidence interval (CI): 1.1–1.3; Table 2]. Specifically, each one-unit increase in NPAR was associated with a 20% higher risk of perioperative MACE. When NPAR was analyzed as a categorical variable (tertiles) in a sensitivity analysis, the risk of perioperative MACE in the highest tertile (tertile 3) was 2.3 times greater than that in the lowest tertile (tertile 1) (OR: 2.3; 95% CI: 1.0–5.1). The relationship between NPAR and perioperative MACE risk was further examined using smooth curve fitting and threshold effect analysis (Figure 2), which revealed a J-shaped association. A two-piecewise linear regression model confirmed a threshold effect at an NPAR value of 13.7 (*p* = 0.005; Table 3). Analysis using complete case data showed a stepwise increase in perioperative MACE risk with rising NPAR levels (Supplementary Table S1). Similarly, our study identified an inverse relationship between eGFR and perioperative MACE risk. Notably, this association remained significant in Model 3 after adjusting for multiple covariates (OR = 0.986; 95% CI: 0.972–0.999; Supplementary Table S2). To explore the underlying mechanism, we conducted a mediation analysis to assess whether eGFR mediated the association between NPAR and perioperative MACEs. After adjusting for age, sex, BMI, hypertension, diabetes, and stroke, the mediation effect remained significant, with approximately 8.4% of NPAR's impact on perioperative MACE being mediated through eGFR levels (Supplementary Table S3). Variance inflation factor (VIF) values for all covariates ranged from 1.07–3.82, indicating no evidence of multicollinearity.

**Table 2:** Association between the NPAR and perioperative MACEs by multiple logistic models.

Exposure	Model 1	Model 2	Model 3
NPAR	1.2 (1.1, 1.3) <0.001	1.2 (1.1, 1.2) <0.001	1.2 (1.1, 1.3) 0.001
NPAR tertile			
Low	1	1	1
Middle	1.2 (0.6, 2.6) 0.570	1.0 (0.5, 2.2) 0.902	0.9 (0.4, 2.0) 0.738
High	5.1 (2.8, 9.4) <0.001	3.5 (1.8, 6.6) <0.001	2.3 (1.0, 5.1) 0.039
*P* for trend	<0.001	<0.001	0.017

Model 1 adjust for: None.

Model 2 adjust for: Age; Gender; BMI; Hypertension; Diabetes.

Model 3 adjust for: Age; Gender; BMI; Hypertension; Diabetes; Stroke; Dialysis; COPD; Ischemic Heart Disease; Myocardial Infarction; Heart Failure; Atrial Fibrillation; Valvular Heart Disease; PTCA; CABG; Hemoglobin; Platelet; Fasting Blood Glucose; Triglyceride; Total Cholesterol; HDL; LDL; Total Bilirubin; Direct Bilirubin; Indirect Bilirubin; TSP; GLB; ALT; AST; ALP; GGT; Creatinine; Uric Acid; eGFR; Potassium; Sodium; Chlorine; tCa; PT; APTT; Fibrinogen; D-dimer; ASA Class; General Anesthesia; Insulin; DOS.

**Table 3:** Threshold effect analysis of NPAR on perioperative MACEs.

perioperative MACEs	Adjusted OR (95% CI), *P*-value
Total, per 1 SD	1.2 (1.1, 1.3) 0.001
Inflection point	13.7
NPAR < 13.7, per 1 SD	0.7 (0.5, 1.0) 0.028
NPAR > 13.7, per 1 SD	1.2 (1.1, 1.4) < 0.001
*P* for log likelihood ratio test	0.005

Adjusted for: Age; Gender; BMI; Hypertension; Diabetes; Stroke; Dialysis; COPD; Ischemic Heart Disease; Myocardial Infarction; Heart Failure; Atrial Fibrillation;Valvular Heart Disease; PTCA; CABG; Hemoglobin; Platelet; Fasting Blood Glucose; Triglyceride; Total Cholesterol; HDL; LDL; Total Bilirubin; Direct Bilirubin;.

Indirect Bilirubin; TSP; GLB; ALT; AST; ALP; GGT; Creatinine; Uric Acid; eGFR; Potassium; Sodium; Chlorine; tCa; PT; APTT; Fibrinogen; D-dimer; ASA Class; General Anesthesia; Insulin; DOS.

## ROC analysis and the incremental predictive value of NPAR

**Figure 3:**
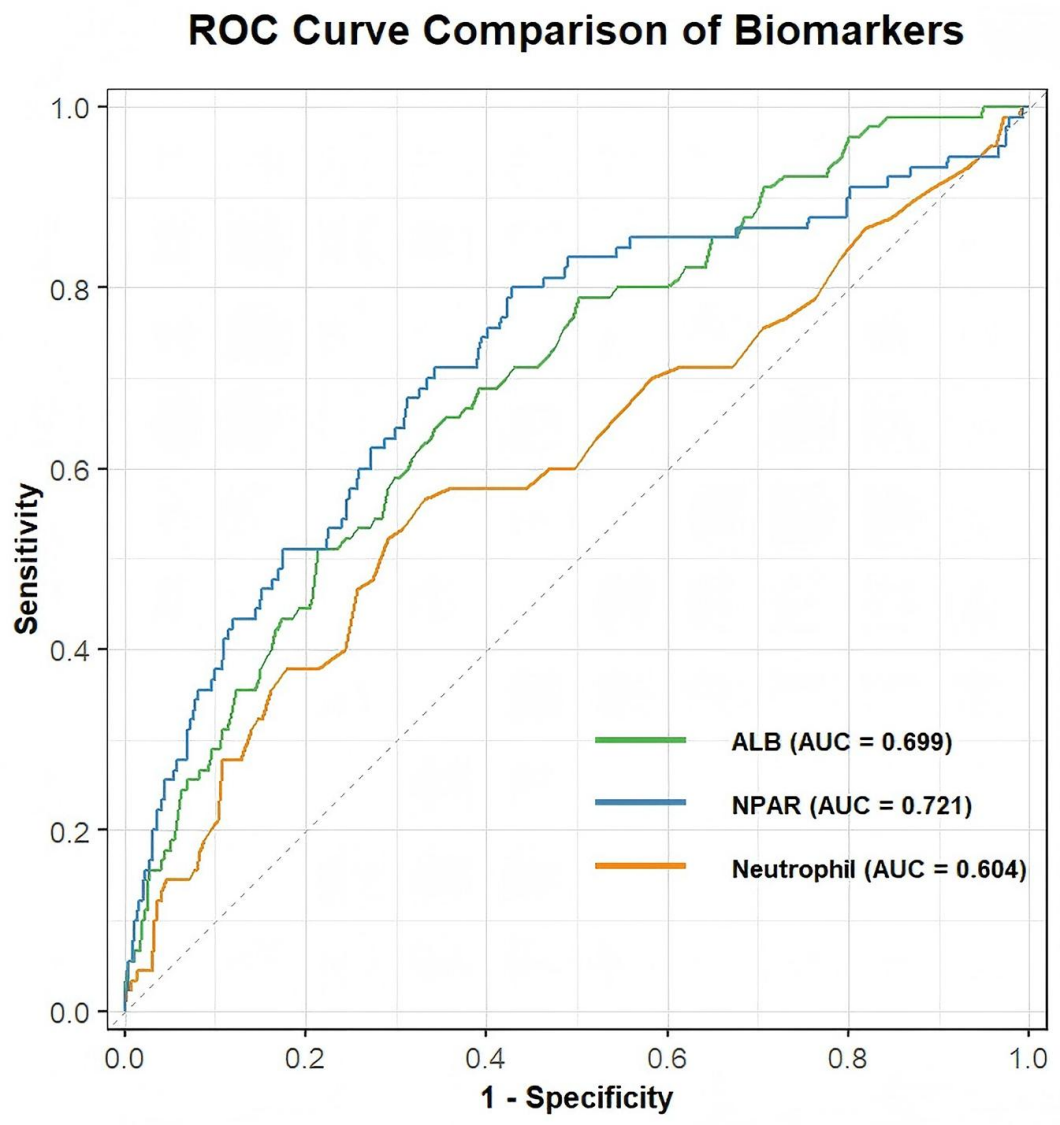
Comparison of predictive performances of NPAR, albumin and neutrophil determined by ROC curves in predicting perioperative MACEs.

**Figure 4:**
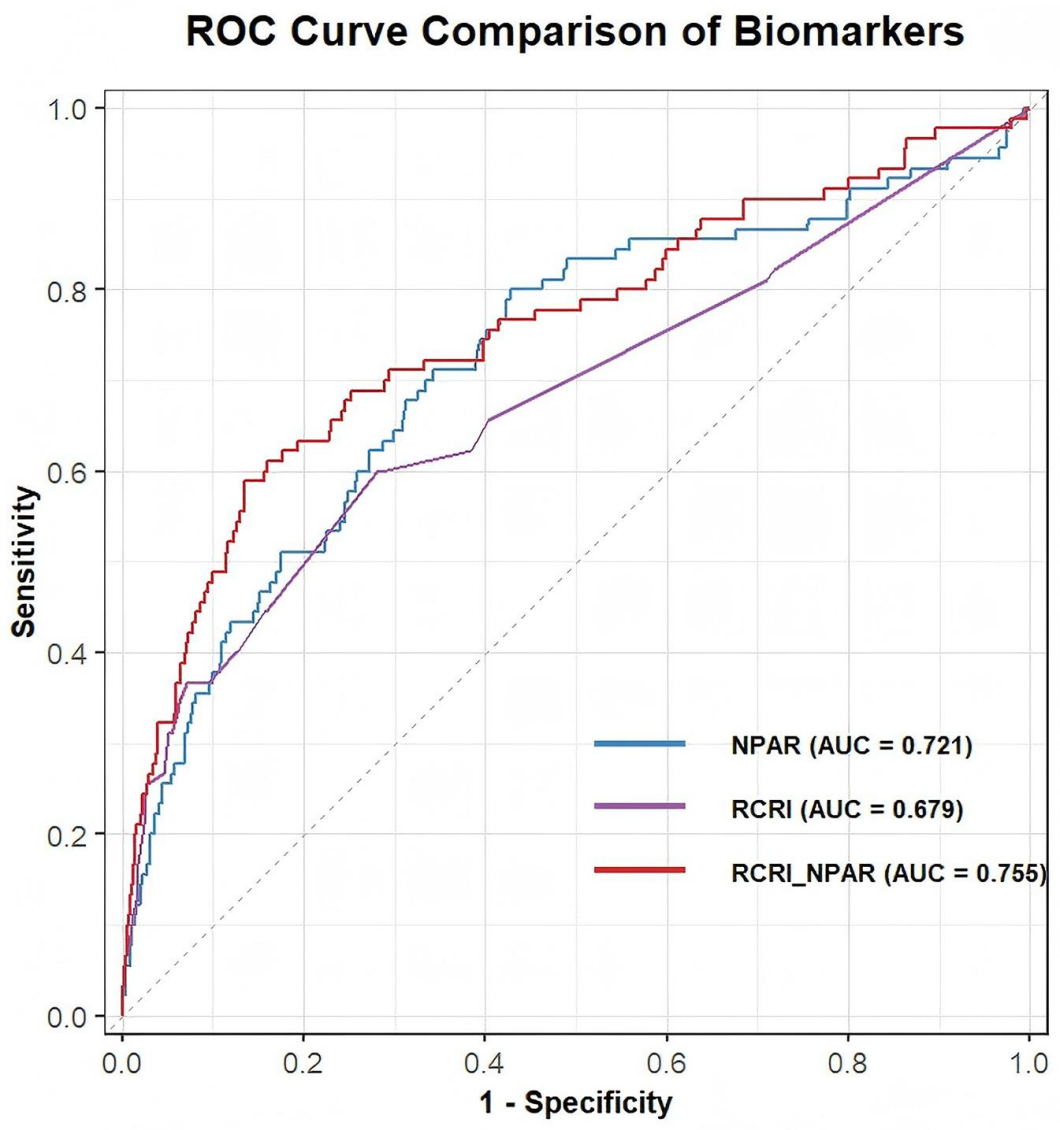
Comparison of ROC curves between NPAR, RCRI models and RCRI model + NPAR for predicting perioperative MACEs.

Figure 3 illustrates the ROC curves and corresponding AUC values for predicting perioperative MACEs. The results show that NPAR achieved an AUC of 0.721, outperforming other markers such as albumin (AUC: 0.699) and neutrophil count (AUC: 0.604). This suggests that NPAR may serve as a more effective predictor of perioperative MACE. As shown in Table 4 and Figure 4, incorporating NPAR into the predictive model significantly improved its performance. Specifically, the addition of NPAR to the RCRI model increased the AUC from 0.679– 0.755, with a statistically significant difference confirmed by DeLong's test (Supplementary Table S4). This improvement translates into one additional high-risk patient being correctly reclassified for every 13 patients evaluated.

**Table 4:** Comparative ROC analysis of NPAR, neutrophil, albumin, RCRI and combined models.

Model	AUC	CI_low	CI_upp	Best_threshold	Specificity	Sensitivity
RCRI	0.679	0.613	0.745	0.040	0.720	0.600
NPAR	0.721	0.661	0.782	0.040	0.573	0.800
Neutrophil	0.604	0.537	0.671	0.050	0.669	0.567
ALB	0.699	0.644	0.754	0.050	0.647	0.656
NPAR + RCRI	0.755	0.695	0.815	0.070	0.860	0.590

**Figure 5:**
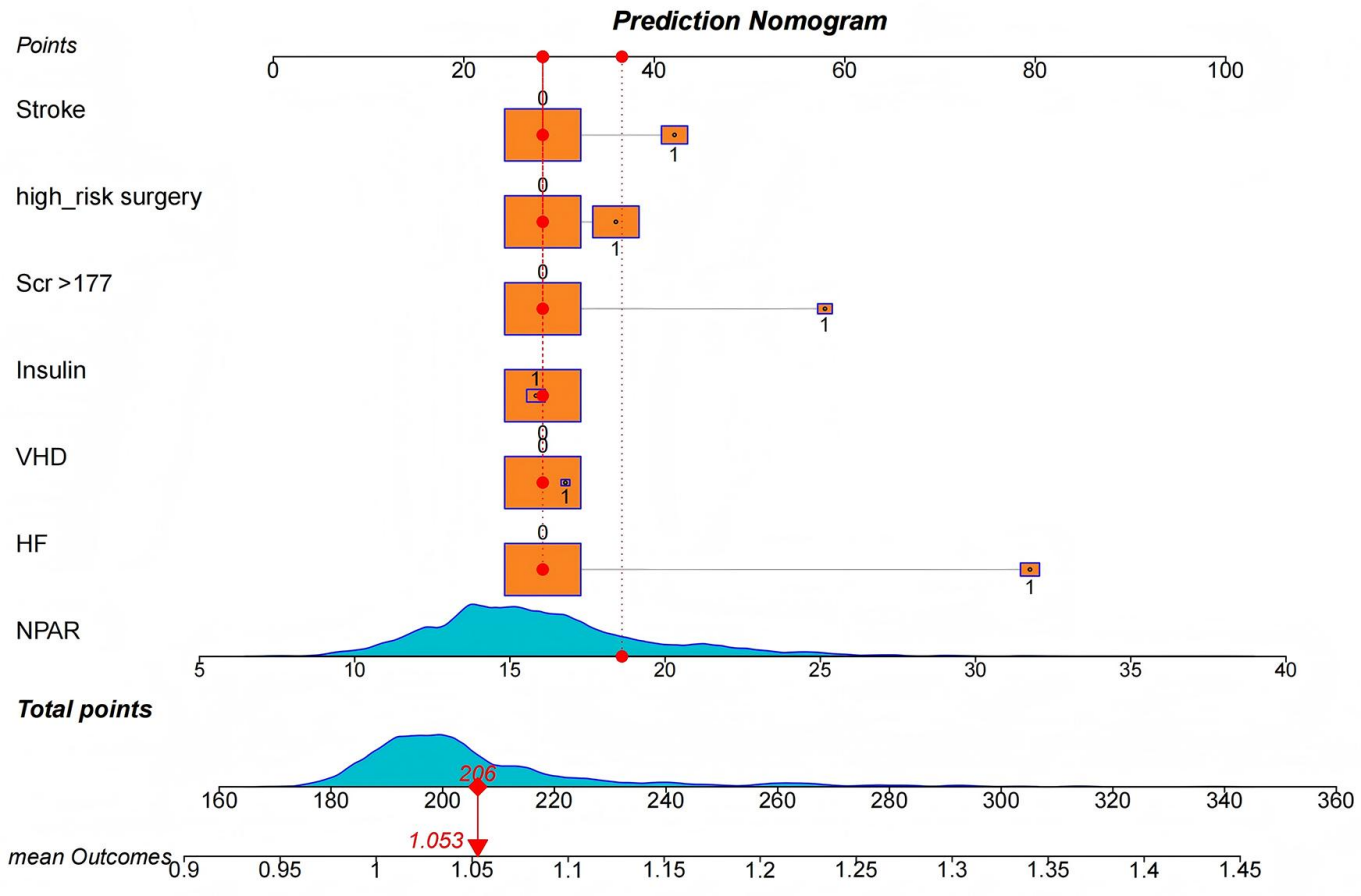
Nomograms, adjusted for RCRI and NPAR, are used to predict perioperative MACEs in patients with SCAD.

The C-statistic, a key indicator of model discrimination, also improved when NPAR was added as a continuous variable to the RCRI model, demonstrating enhanced predictive power both statistically and clinically (Table 5). Model fit was also significantly improved, with a continuous NRI of 0.599 (95% CI: 0.311–0.802, *p* < 0.001) and an IDI of 0.035 (95% CI: 0.013–0.057, *p* = 0.002), underscoring the practical utility of these enhancements. In order to better apply NPAR in clinical practice, we built a nomogram (Figure 5) that combines NPAR and RCRI model to predict perioperative adverse cardiovascular events in patients with stable coronary heart disease, so that it can be used by clinicians. The calibration curve (Supplementary Figure S2) closely aligned with the reference line, indicating strong model calibration and predictive performance. Additionally, DCA (Supplementary Figure S3) demonstrated a meaningful net benefit, highlighting the model's potential to support clinical decision-making and real-world application.

**Table 5:** Added predictive ability and reclassification statistics of the NPAR.

Model	C-statistic(95% CI)	*P* value	Continuous NRI(95% CI)	*P* value	IDI(95% CI)	*P* value
RCRI	0.679 (0.613, 0.745)		Reference		Reference	
RCRI + NPAR	0.755 (0.645, 0.815)	0.005	0.599 (0.311,0.802)	<0.001	0.035 (0.013,0.057)	0.002

### Establishment and validation of the prediction model

**Figure 6:**
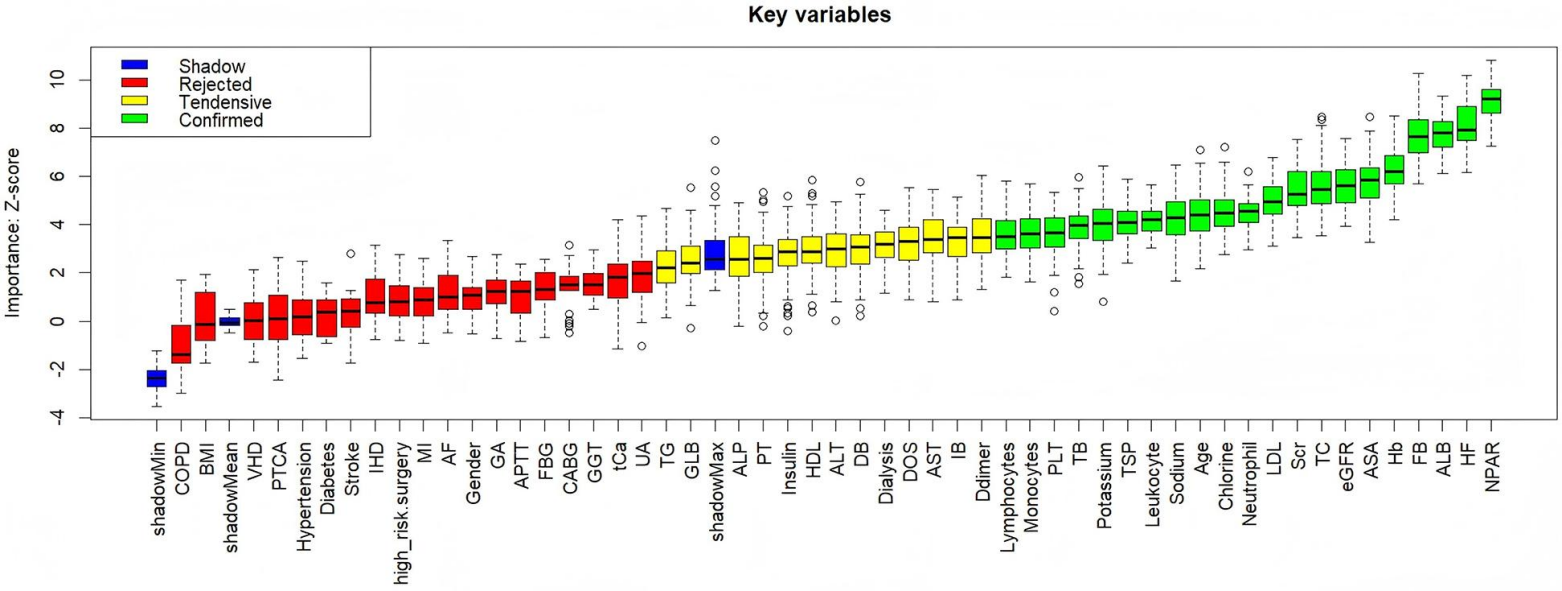
Feature selection based on Boruta algorithm. The horizontal axis displays the name of each variable, and the vertical axis represents the Z-score of each variable.

**Figure 7:**
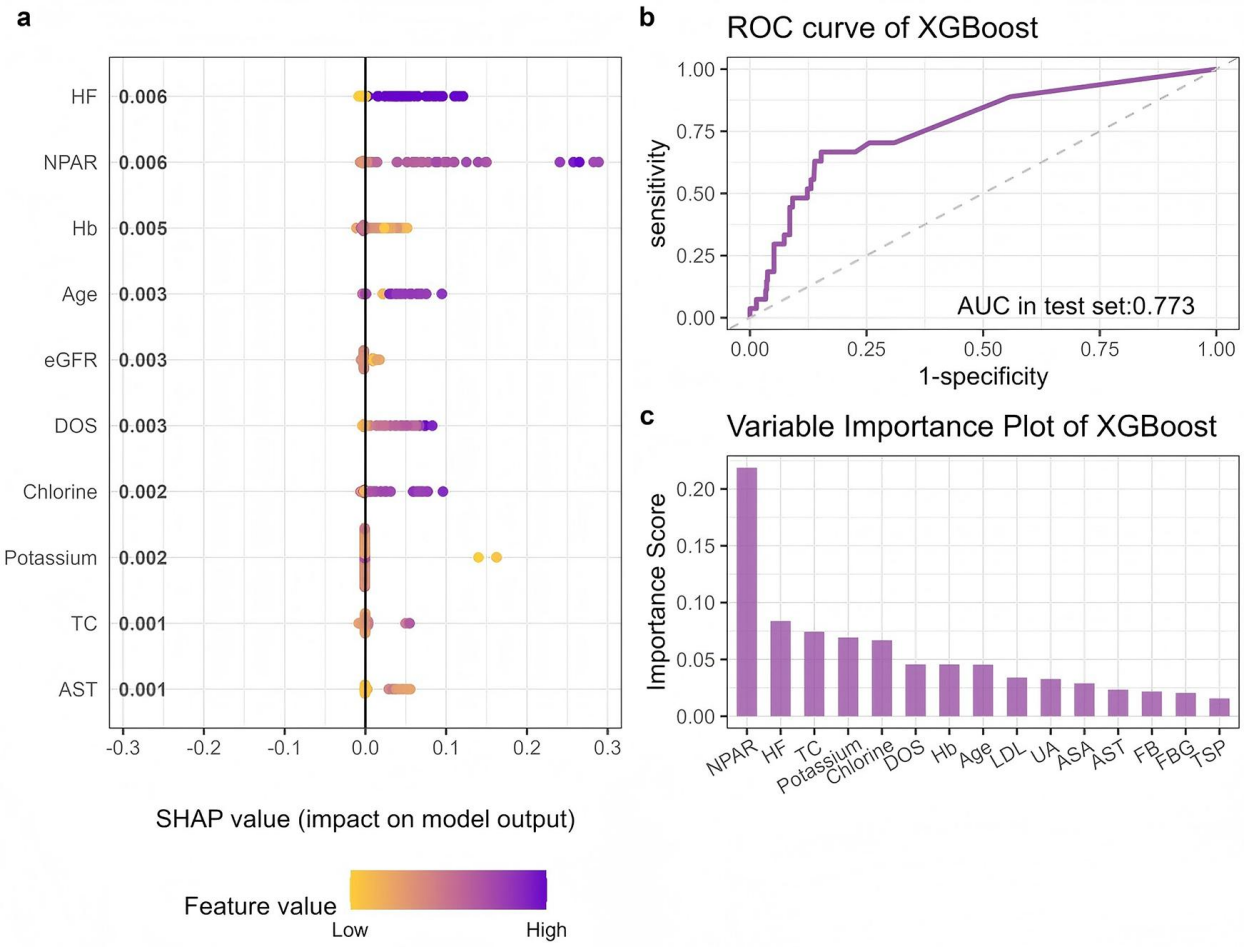
XGBoost model predicts perioperative MACEs: **(a)** distribution of the impact each feature had on the full model output using SHAP values, **(b)** ROC curve of XGBoost, **(c)** Variable importance ranking.

Figure 6 presents the results of feature selection based on the Boruta algorithm. Variables identified as important (shown in the green area) were incorporated into the development of the machine learning model. Supplementary Table S5 lists the hyperparameters and the optimal parameter settings for the XGBoost model. Figure 7 displays the ROC curve for the XGBoost model, with an AUC of 0.773. To enhance the interpretability of the model output, we applied SHAP analysis to identify the variables most strongly associated with MACEs as determined by the XGBoost model. The bar plot ranks the key variables in descending order of importance. SHAP values provide an effective method to reveal the features contributing to individual patient predictions and to quantify the impact of each variable on MACEs prediction. Among the features, NPAR emerged as a particularly important predictor.

## Discussion

Given the higher perioperative MACE risk in patients with SCAD compared to the general population, preoperative cardiovascular risk assessment in this high-risk group is critical for optimizing risk reduction strategies and improving surgical outcomes (30). This study investigated the association and predictive value of the NPAR in assessing perioperative MACEs in patients with stable coronary artery disease undergoing NCS. Our results demonstrated that elevated preoperative NPAR was significantly associated with an increased risk of perioperative MACEs. A J-shaped relationship was observed between NPAR and MACEs, with the risk rising notably when NPAR exceeded 13.7. Moreover, mediation analysis suggested that decreased eGFR may be one of the underlying mediators. ROC analysis and machine learning models further indicated that incorporating NPAR improved the predictive accuracy for perioperative MACEs. We have also constructed an effective perioperative predictive nomogram, and the perioperative risk probability of MACEs in patients with SCAD can be estimated by calculating scores. These findings suggest that NPAR may serve as a key predictor of adverse cardiovascular outcomes during the perioperative period.

As key components of the inflammatory response, neutrophils not only contribute to the development of coronary atherosclerosis but also play a critical role in atherothrombotic events, adverse remodeling, mortality, and the occurrence of MACEs following ACS (31, 32). Albumin has traditionally been regarded as a marker of nutritional status. It is well established that low serum albumin levels reflect the severity of inflammation and contribute to the progression of cardiovascular diseases (CVDs) (33). Furthermore, hypoalbuminemia has been associated with increased mortality and poor prognosis in patients with myocardial infarction (34). Physiological stress responses — including heightened sympathetic nervous system activity, catecholamine release, and hemodynamic alterations—can be triggered by surgery. Surgical trauma also induces a systemic inflammatory response, which may increase myocardial oxygen demand and accelerate myocardial injury, particularly in susceptible individuals (12). As a combination of two inflammation-related markers, NPAR reflects both nutritional status and the degree of inflammation. Some studies have suggested that NPAR may provide a more comprehensive assessment of inflammatory responses (35, 36). Mechanistically, perioperative sterile inflammation activates neutrophils and promotes the formation of neutrophil extracellular traps (NETs), which disrupt the endothelial glycocalyx, reduce nitric oxide bioavailability, and impair microcirculation. At the same time, inflammation lowers serum albumin, diminishing its antioxidant and endothelial-protective effects. By combining increased neutrophil percentage with decreased albumin into a single ratio, NPAR may better capture this inflammation endothelium microcirculation pathway, which could explain its stronger prognostic value compared with either marker alone.

Activation of neutrophils and the release of inflammatory mediators such as TNF-α and interleukin-6 (IL-6) contribute to vascular endothelial injury, exacerbate vascular dysfunction, and promote atherosclerosis (37). This inflammatory cascade leads to elevated NPAR levels. In addition, oxidative stress, through the generation of reactive oxygen species (ROS), further damages cellular structures, worsens vascular health, and perpetuates the vicious cycle of inflammation. Chronic inflammation has also been shown to impair albumin synthesis, thereby further compromising patient health (38). In recent years, studies have shown that NPAR is an inflammation-based prognostic predictor for various cardiovascular diseases, including atrial fibrillation, chronic heart failure, acute ST-elevation myocardial infarction (STEMI), and cardiogenic shock (31, 35, 36, 39, 40). For example, a retrospective study by Wang et al. involving 622 patients with heart failure found that patients with higher NPAR levels had significantly increased mortality at 90 days (HR, 95% CI: 2.21, 1.01–4.86), 1 year (HR, 2.13, 95% CI: 1.30–3.49), and 2 years (HR, 2.06, 95% CI: 1.37–3.09), compared with those with lower NPAR levels (*P* for trend < 0.05). They also demonstrated that NPAR not only correlated with all-cause mortality in patients with chronic heart failure but also provided better prognostic value than either albumin or neutrophil percentage alone (35). An analysis based on the MIMIC-III database showed that among 3,106 critically ill patients diagnosed with coronary artery disease, patients in the highest NPAR quartile had significantly higher all-cause mortality at 30 days (HR, 1.924, 95% CI: 1.471–2.516), 90 days (HR, 2.053, 95% CI: 1.646–2.560), and 365 days (HR, 2.063, 95% CI: 1.717–2.480) compared to those in the lowest quartile (34). In a study by Cui et al., NPAR was identified as an independent predictor of in-hospital mortality among STEMI patients (41). Additionally, two studies involving patients undergoing peritoneal dialysis consistently found that higher NPAR levels were independently associated with increased risks of all-cause and cardiovascular mortality (42, 43). Among critically ill patients, elevated NPAR levels at admission were also independently associated with higher all-cause mortality and prolonged hospital stay (44). Moreover, a previous cross-sectional study demonstrated that elevated NPAR levels were associated with a higher prevalence of chronic kidney disease (CKD), lower eGFR, and increased proteinuria, with these associations consistently observed across different population settings (27). Given that impaired renal function is an established risk factor for MACEs, these findings suggest that eGFR may serve as a potential mediator in this relationship. Our study yielded similar results, supporting this hypothesis. Furthermore, although eGFR only accounted for 8.4% of the NPAR–MACE association—indicating a limited role as a single mediator—the perioperative inflammation–endothelium–microcirculation axis likely also involves cytokines such as IL-6 and TNF-α, which are not routinely measured, as well as surgical trauma intensity and anesthesia modality. Multicenter prospective studies that systematically collect these variables are therefore warranted to further dissect the multifactorial mechanisms by which elevated NPAR drives perioperative cardiovascular events.

Currently, the RCRI is the most commonly used model for predicting perioperative MACE risk. However, recent studies have indicated that RCRI has limited discriminatory ability (45), particularly among patients with known SCAD (46). Incorporating NPAR into the RCRI model may enhance its predictive performance for MACEs. Although RCRI is widely used in NCS, it often lacks the precision required for individualized risk assessment (47, 48). For example, data from the VISION cohort showed that among patients aged 45 years and older, one in twelve individuals without traditional RCRI risk factors still experienced cardiac complications following major non-cardiac surgery (49). Many studies have explored the use of biomarkers to improve the predictive ability of RCRI in the non-cardiac surgical setting. The value of brain natriuretic peptide (BNP) in preoperative risk assessment and cardiac troponin I (cTnI) in detecting prognostically relevant myocardial ischemia has been well established (50). Compared to traditional cardiac biomarkers, NPAR may be a more suitable preoperative screening tool due to its cost-effectiveness and broad applicability. NPAR offers a promising alternative for personalized risk assessment, potentially identifying high-risk patients who might otherwise be overlooked. The combination of the XGBoost classifier and the Boruta algorithm has been widely applied across various fields, including medicine, computer science, molecular biology, and economics, demonstrating superior overall model performance compared to other approaches (51–54). In medicine, this approach has been used not only for disease diagnosis and prognosis but also for biomarker discovery and risk prediction (51, 54). Both the Boruta algorithm and SHAP analysis in our study identified NPAR as an important predictor of perioperative MACE risk. To our knowledge, this is the first study to investigate the association between NPAR and perioperative MACEs in patients with SCAD, as well as to evaluate the prognostic value of integrating NPAR into the RCRI model. NPAR may serve as a reliable, inexpensive, and easily accessible biomarker for assessing the risk of perioperative MACEs in SCAD patients. By utilizing NPAR for MACE risk assessment, our study provides a practical tool to help clinicians identify high-risk patients early, guiding preventive strategies and timely interventions in clinical practice. Routine monitoring of inflammatory markers such as neutrophil counts and albumin levels could facilitate early detection of high-risk individuals and timely intervention. Furthermore, exploring the molecular mechanisms underlying the association between NPAR and perioperative MACEs in SCAD patients may offer insights into potential therapeutic targets. This study was conducted at a single center, limiting the ability to fully eliminate biases related to patient selection, regional healthcare differences, and variations in follow-up management. As a result, the generalizability of the findings is restricted. Additionally, the low number and incidence of outcome events may affect the stability of the multivariable models and the generalization performance of the predictive models. Although the machine learning models demonstrated good performance in internal cross-validation, they have yet to be validated in independent external cohorts or prospective studies. Further external validation is needed to confirm the robustness and clinical applicability of the models. Despite constructing multiple adjusted models and controlling for numerous covariates, inherent unmeasured confounding factors associated with the retrospective design may still impact the authenticity of the results. Moreover, while our mediation analysis identified eGFR as a partial mediator in the association between NPAR and perioperative MACE, the proportion mediated was relatively modest (8.4%), suggesting that other unmeasured pathophysiological pathways—such as those involving inflammatory cytokines (e.g., IL-6, TNF-α), intensity of surgical stress, or specific anesthesia techniques—may contribute more substantially to this relationship. The absence of systematically collected data on these potential mediators represents an important limitation of our study. In addition, the relatively short follow-up period may not have fully captured the long-term prognostic impact of NPAR, and the presence of some missing data could have introduced potential bias despite our efforts at statistical adjustment. In summary, before incorporating NPAR into clinical decision-making, multicenter prospective studies are required, with routine postoperative monitoring of inflammatory and myocardial injury markers and longer follow-up periods, to externally validate the models and further assess their applicability across different populations and healthcare settings.

## Conclusion

This study identified the NPAR as an independent predictor of MACE during the perioperative period in patients with stable coronary artery disease undergoing NCS. Elevated preoperative NPAR levels were significantly associated with an increased risk of MACE, with eGFR potentially acting as a mediator in this association. Integrating NPAR into established risk assessment models—particularly the RCRI—substantially improved predictive performance. Additionally, the development of a nomogram allows for individualized risk estimation, facilitating clinical decision-making. The prognostic value of NPAR was further validated through machine learning algorithms, reinforcing its potential role in perioperative risk stratification. These findings provide a strong foundation for future prospective cohort studies and mechanistic investigations to confirm the clinical utility and pathophysiological relevance of NPAR in this setting.

## Data Availability

The original contributions presented in the study are included in the article/Supplementary Material, further inquiries can be directed to the corresponding author.
