# Supplementary material for: Association and predictability of major perioperative cardiovascular adverse events and elevated neutrophil percentage-to-albumin ratio in patients with stable coronary artery disease undergoing non-cardiac surgery
Source: Front Cardiovasc Med. 2025 Sep 19;12:1623731. doi: 10.3389/fcvm.2025.1623731 (PMC12491289; doi:10.3389/fcvm.2025.1623731)
Supplement: Supplementary file 2 [file Datasheet2.pdf]

Table S1 Association between the NPAR and perioperative MACEs by multiple logistic models（unimputed data）

Exposure	Non-adjusted	Adjust I	Adjust II
**NPAR**	1.2 (1.1, 1.3) <0.001	1.2 (1.1, 1.2) <0.001	1.2 (1.0, 1.3) 0.006

Model 1 adjust for: None

Model 2 adjust for: Age; Gender; BMI; Hypertension; Diabetes

Model 3 adjust for: Age; Gender; BMI; Hypertension; Diabetes; Stroke; Dialysis; COPD; Ischemic Heart Disease; Myocardial Infarction; Heart Failure; Atrial Fibrillation; Valvular Heart Disease; PTCA; CABG; Hemoglobin; Platelet; Fasting Blood Glucose; Triglyceride; Total Cholesterol; HDL; LDL; Total Bilirubin; Direct Bilirubin; Indirect Bilirubin; TSP; GLB; ALT; AST; ALP; GGT; Creatinine; Uric Acid; eGFR; Potassium; Sodium; Chlorine; tCa; PT; APTT; Fibrinogen; D-dimer; ASA Class; General Anesthesia; Insulin; DOS

Table S2 Association between the eGFR and perioperative MACEs by multiple logistic models

Exposure	Non-adjusted	Adjust I	Adjust II
**eGFR**	0.981 (0.974, 0.989) <0.01	0.985 (0.977, 0.993) <0.01	0.986 (0.972,0.999) 0.045

Non-adjusted model adjust for: None

Adjust I model adjust for: Age; Gender; BMI; Hypertension; Diabetes; Stroke

Adjust II model adjust for: Age; Gender; BMI; Hypertension; Diabetes; Stroke; Dialysis; COPD; Ischemic Heart Disease; Myocardial Infarction; Heart Failure; Atrial Fibrillation; Valvular Heart Disease; PTCA; CABG; Hemoglobin; Platelet; Neutrophil;Fasting Blood Glucose; Triglyceride; Total Cholesterol; HDL; LDL; Total Bilirubin; Direct Bilirubin; Indirect Bilirubin; TSP; ALB; GLB; ALT; AST; ALP; GGT; Creatinine; Uric Acid; Potassium; Sodium; Chlorine; tCa; PT; APTT; Fibrinogen; D-dimer; ASA Class; General Anesthesia; Insulin; DOS

Table S3 Mediation analysis of the association between NPAR and Perioperative MACEs mediated by eGFR

	Total effect	Direct effect	Mediation effect	Propotion mediated
**Estimate (95% CI)**	0.026 (0.018, 0.034)	0.024 (0.016, 0.032)	0.002 (0.001, 0.004)	0.084 (0.019, 0.173)
**P-value**	<0.01	<0.01	＜0.01	＜0.01

Adjusting variables: Age; Gender; BMI; Hypertension; Diabetes; Stroke

Table S4 Delong test for ROC curves

Comparison	AUC_Model1	AUC_Model2	Z_Value	P_Value
**NPAR+RCRI vs RCRI**	0.755	0.679	2.841	0.005
**NPAR+RCRI vs NPAR**	0.755	0.721	1.767	0.077
**NPAR vs RCRI**	0.721	0.679	1.069	0.285

Table S5 Hyperparameter of the XGBoost model

Model	Hyperparameter	Tuning scope	Optimal hyperparameter
**XGBoost**	n_estimators	(100, 1000)	100
	max_depth	(3, 10)	4
	learning_rate	(0.01, 0.3)	0.01
	subsample	(0.5, 1)	0.8
	colsample_bytree	(0.5, 1)	0.8

Figure S1 Missing data ratio

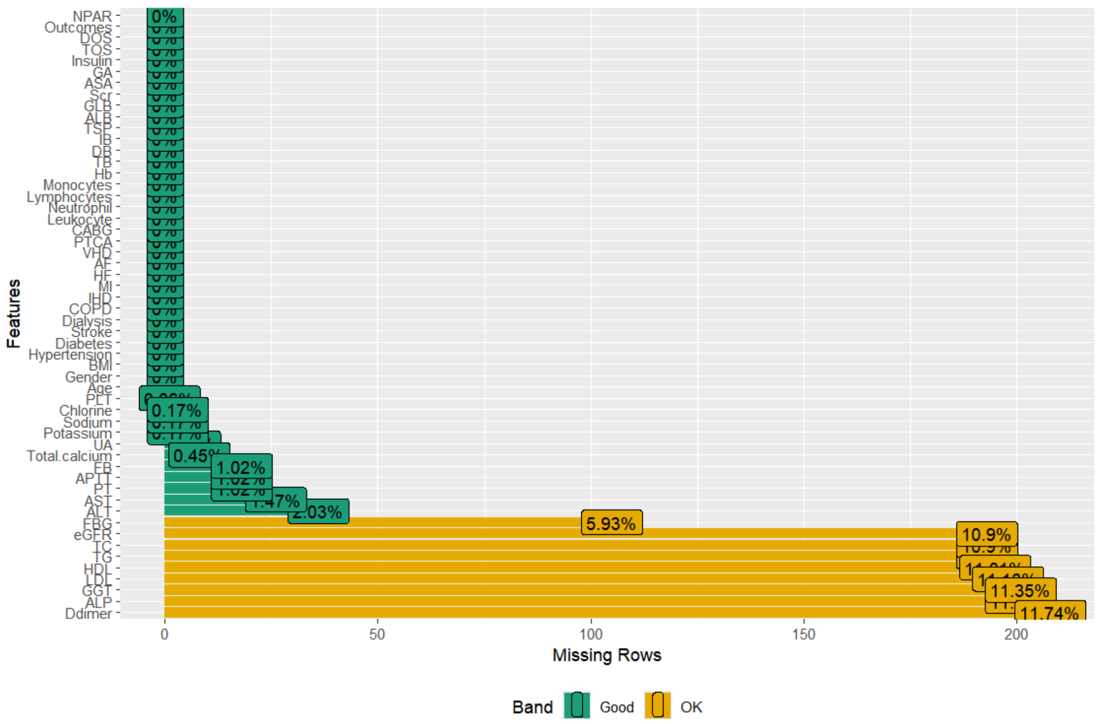

Figure S2

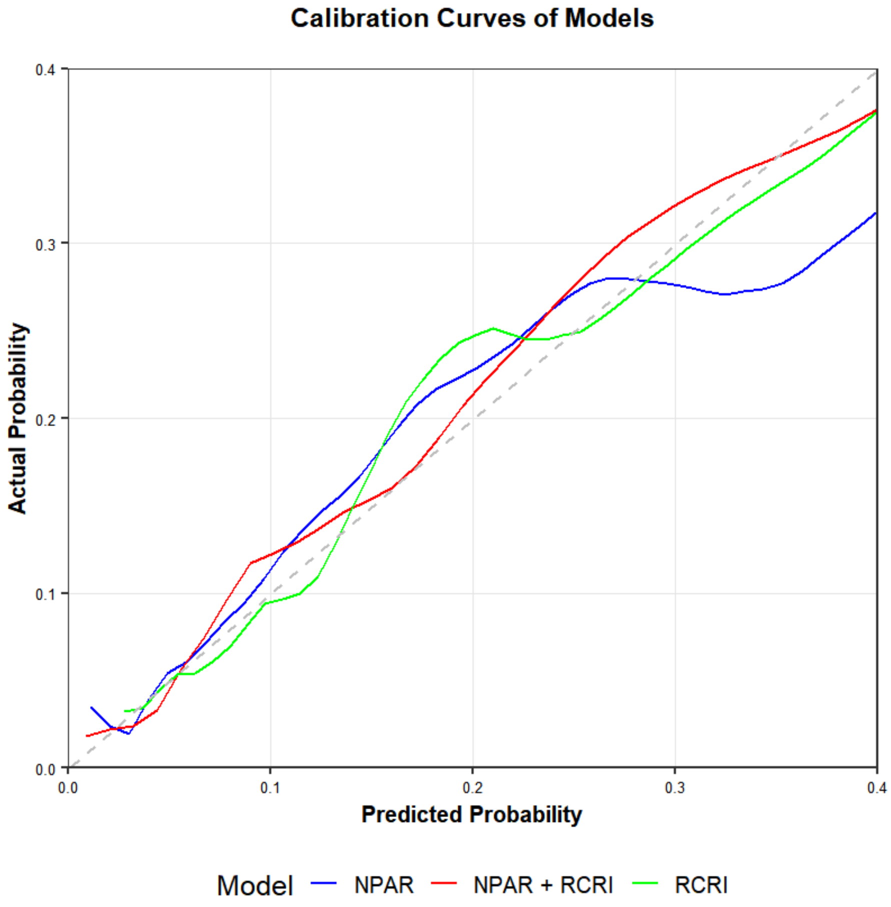
Calibration Curves of Models
Actual Probability
Predicted Probability
0.0
0.1
0.2
0.3
0.4
Model
NPAR
NPAR + RCRI
RCRI

Figure S3

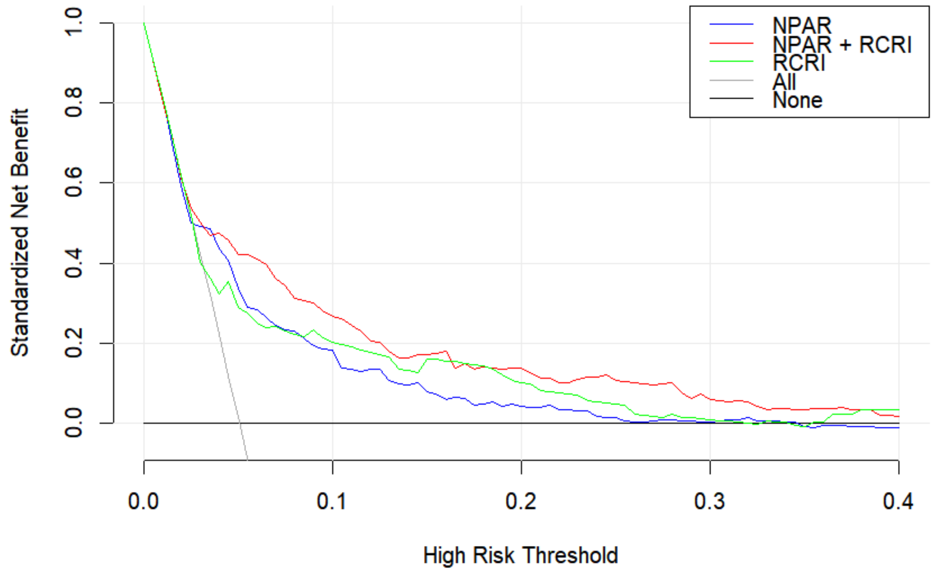
NPAR
NPAR + RCRI
RCRI
All
None
Standardized Net Benefit
0.0
0.2
0.4
0.6
0.8
1.0
0.0
0.1
0.2
0.3
0.4
High Risk Threshold
